# Individual variability in finger-to-finger transmission efficiency of *Enterococcus faecium* clones

**DOI:** 10.1002/mbo3.156

**Published:** 2014-01-02

**Authors:** Rosa del Campo, Ana María Sánchez-Díaz, Javier Zamora, Carmen Torres, Luis María Cintas, Elvira Franco, Rafael Cantón, Fernando Baquero

**Affiliations:** 1 Servicio de Microbiología, Hospital Universitario Ramón y CajalMadrid, Spain; 2 Instituto Ramón y Cajal de Investigación Sanitaria (IRYCIS)Madrid, Spain; 3 Unidad de Bioestadística Clínica, Hospital Universitario Ramón y CajalMadrid, Spain; 4 CIBER en Epidemiología y Salud Pública (CIBERESP)Spain; 5 Área Bioquímica y Biología Molecular, Universidad de La RiojaLogroño, Spain; 6 Departamento de Nutrición, Bromatología y Tecnología de los Alimentos, Facultad de Veterinaria, Universidad Complutense de MadridMadrid, Spain; 7 Departamento de Farmacia y Tecnología Farmacéutica, Facultad de Farmacia, Universidad Complutense de MadridMadrid, Spain

**Keywords:** Bacterial transmission, enterococcal clones, hand hygiene

## Abstract

A fingertip-to-fingertip intraindividual transmission experiment was carried out in 30 healthy volunteers, using four MLST-typed *Enterococcus faecium* clones. Overall results showed an adequate fit goodness to a theoretical exponential model, whereas four volunteers (13%) exhibited a significantly higher finger-to-finger bacterial transmission efficiency. This observation might have deep consequences in nosocomial epidemiology.

## Introduction

Few works in Medicine have had the interventional impact of the book “Etiology, Concept and Prophylaxis of Childbed Fever” by Ignaz Philipp Semmelweis, published in 1847. More than 165 years later, hand hygiene remains a general measure that significantly contributes to the prevention and control of communicable diseases; in healthcare settings, improved hand hygiene practices reduce cross-transmission of multidrug-resistant microorganisms, prevent healthcare-associated infections, and save costs (Stewardson et al. 2011; Monnet and Sprenger 2012). Surprisingly, during these 165 years, very little has been done to investigate the biological basis underlying the process of bacterial transmission by hands, and particularly from the side of possible variations among individual hosts. In this study, we explored the efficiency in finger-to-finger transmission of bacteria, which may eventually be variable, in relation with different human individuals and bacterial clones. Certainly, this observation reinforces the classic historical links between microbiology, microbial ecology, and the epidemiology of infectious diseases.

## Material and Methods

The design of the study was focused to detect possible interindividual differences by testing finger-to-finger transmission within the same individual, in comparison with other individuals. *Enterococcus faecium* was chosen as the experimental organism due to both its intestinal carriage and its capacity to produce hospital outbreaks, in which hand-to-hand transmission seems to be a critical event (Noskin et al. 1995; Duckro et al. 2005; Willems and van Schaik 2009).

We explored the possible variability in intraindividual bacterial transmission in 30 healthy young adults (20 females, 10 males, ranging 23–50 years), mostly fellows and technicians of our Department, who served as volunteers (with informed consent). Volunteers were not exposed to antimicrobial compounds (including alcohol preparations) systemically or locally (last hand wash with nonantibacterial liquid soap (Kriss 5.5; Tein S.L. Madrid, Spain), at least 90 min before sampling) or to harsh chemicals, such as acids, bases, and solvents, and were examined to ensure hands were free of clinically evident dermatoses or any other lesions. Four Multi-Locus Sequence Typing (MLST)-characterized *E. faecium* clones were included in the study: (1) ST18-CC17 *E. faecium* H182, cause of a nosocomial outbreak (Freitas et al. 2009); (2) ST203-CC17 *E. faecium* RYC49, isolated from a bacteremia in a community-patient; (3) ST315-CC17 bacteriocin-producer *van*A-*E. faecium* RC714, obtained from a human intestinal colonization sample (del Campo et al. 2001); and (4) ST178-CC94 *E. faecium* L50, a multiple bacteriocin-producing strain isolated from a Spanish dry-fermented sausage (Cintas et al. 2000).

All four clones were incubated separately in 10 mL of Brain-Heart Infusion (BHI) overnight at 37°C, and then adjusted by optical density (previously tested with colony-forming units, [CFU] counts) to obtain a cell concentration of 10^9^ CFU/mL. An aliquot of 10 *μ*L of each one of the clones (˜10^7^ cells) was gently deposited and spread on both thumb tip surfaces (˜130 mm diameter, defined by four-points marks in the skin) of each volunteer. The initial bacterial load on the thumbs was assessed in the following way: after complete drying, the 100-mm-diameter mouth of an eppendorf plastic tube containing 750 *μ*L of sterile saline solution at 37°C was pressed on the contaminated thumb surface (sampling surface: 0.78 cm^2^), and vigorously shaken five times to allow bacterial cells to be suspended into the saline. To explore finger-to-finger transmission, the second contaminated thumb was put in close static contact (assuring full surface contact, but with minimal pressure and preventing twists or wipes) for 10 sec with the index fingertip of the other hand of the same individual. Bacteria were recovered from the index fingertip by washing after application of a new eppendorf plastic tube (see above). Subsequently, the contaminated index fingertip was put in contact with the middle fingertip of the opposite hand, and the bacteria from the index finger were collected with another eppendorf tube following the previously described procedure. Finally, the middle fingertip was pressed on the ring finger of the other hand, and bacteria were again collected. All saline bacterial suspensions were immediately mixed in 1 mL of soft agar and seeded onto M-*Enterococcus* agar and incubated 24 h at 37°C for bacterial counting. After the experiment, fingertips were thoroughly washed with the same liquid soap and running water and then disinfected by firmly rubbing with an alcohol preparation. The experiment was separately repeated four times per enterococcal clone and human individual along six consecutive months.

Bacterial counts into M-*Enterococcus* medium showed a consistent recovery of about (˜10^6^ cells) in the control thumb immediately (after drying time) of inoculation. An exponential decay of CFUs obtained in the sequential finger-to-finger transfers occurs in most volunteers; the transition from finger-to-finger produced typically a decay of 1.5 log in counts (decay is composed by real transmission bottleneck plus sampling-culture bacterial loss). Similar decay kinetics was found in transfer experiments from hands to surfaces in *Escherichia coli* (Lingaas and Fagernes 2009). The most frequent case in volunteers was counts of less than 10 CFUs after the third fingertip-to-fingertip transfer and no CFU recovery in the last (fourth) transfer. Unexpectedly, in a proportion of volunteers, counts of 10^3^ and 10^2^ CFUs were consistently obtained during the third and fourth transfers.

CFU data from the four replicates of every volunteer finger-to-finger transmission experiment were used to fit exponential CFU count decay models, one for each of the four enterococcal clones. The exponential model assumes that the CFU count collected from every next fingertip is proportional to the previous fingertip CFU count. This assumption appears plausible. Goodness-of-fit tests were performed to assess the adequacy of the exponential models produced. The distribution of the exponential decay parameters within every clone was described and checked for normality and for the source of any departure from normal distribution (skewness and kurtosis) (D'Agostino et al. 1990). Between clones, comparison of exponential decay parameters was performed by fitting a population average panel data model by using Generalized Estimating Equations (GEE) assuming interchangeable within-individual correlation structure. Stata software v.11.0 (Stata Corp., College Station, TX) was used for the analysis.

## Results and Discussion

**Figure 1:**
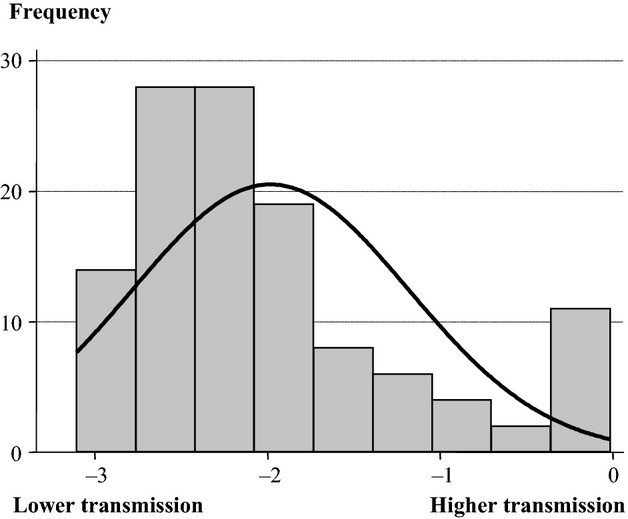
Frequency distribution of exponential decay parameter of all 30 individuals overall the four *Enterococcus faecium* clones. Note that lower decay slopes correspond to lower efficiency in the bacterial transmission.

**Figure 2:**
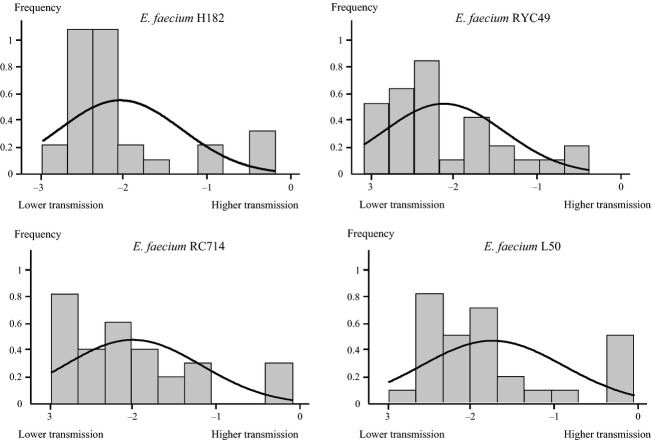
Frequency distribution of exponential decay parameter of all 30 individuals stratified by *Enterococcus faecium* clones.

One-hundred twenty exponential models were fitted for the 30 individuals and the four *E. faecium* clones. The mean, standard deviation, Shapiro-Wilks normality test results, and skewness and kurtosis tests of the exponential decay parameters are presented in Table 1. The frequency distribution of the exponential decay parameter estimated for all individuals and clones combinations clearly showed an asymmetrical right tail containing an overrepresentation of high transmitter individuals with their exponential decay parameters close to zero (Fig. 1). CFU count data from the replications of the finger-to-finger transmission experiments showed adequate goodness of fit to the proposed theoretical exponential model. In fact, only 12 out of the 120 (10%) fitted models showed some lack of fitness with goodness-of-fit test *P*-values greater than 0.05 (Figs. 1, 2). On the contrary, only four models showed lack of fit at 0.1 significance level. All four distributions showed statistical significant departures from normality caused by significant skewness. These results demonstrate that four volunteers among the 30 studied (13%) exhibited a significant higher efficiency in the intraindividual bacterial hand transmission.

**Table 1:** Exponential decay parameter for the *Enterococcus faecium* clones in the intraindividual transmission experiments.

Clone	Mean	SD	Shapiro-Wilks normality test	Kurtosis test	Skewness test
ST18-CC17-H182	−2.02	0.13	0.81 (*P* = 0.0001)	3.95 (*P* = 0.13)	1.39 (*P* = 0.002)
ST203-RYC49	−2.08	0.14	0.92 (*P* = 0.022)	3.00 (*P* = 0.60)	0.88 (*P* = 0.034)
ST315-RC714	−2.03	0.15	0.88 (*P* = 0.002)	3.42 (*P* = 0.31)	1.06 (*P* = 0.013)
ST178-CC94-L50	−1.80	0.16	0.88 (*P* = 0.002)	2.58 (*P* = 0.88)	0.86 (*P* = 0.038)

Regarding the enterococcal clones, the foodborne ST178-CC94 *E. faecium* L50 clone was the most transmissible in comparison with the other grouped in the CC17, especially ST203-*E. faecium* RYC49 clone (*P* = 0.021), and also with ST-18 *E. faecium* H182 (*P* = 0.066) and ST-315 *E. faecium* RC714 (*P* = 0.057) (Fig. 2). We should be aware that the transmission of *E. faecium* CC17 clones is related with the high-density fecal colonization (Ruiz-Garbajosa et al. 2012), not necessarily depending on the parameters studied in this work.

The mechanisms involved in differences among individuals remain to be elucidated. Transepidermal water loss and fingertip temperature measurements were performed in all volunteers using the Tewameter® TM300 apparatus (Courage&Khazaka electronic GmbH, Mathias, Germany), and a digital thermometer PCE-T312. No significant association was found with any of the different transmission patterns. Other possibilities remain to be investigated, such as fatty acid composition of the skin, role of resident bacteria, corneocytes cell shedding, and anatomy of fingerprint crests. We discarded consistent differences among individuals in the finger pressure they exerted, using an ink pad and serial paper fingerprints (after contact of the volunteer's finger to a new ink pad, the finger was pressed by a single investigator on a papers' sheet; with no significant differences in the number of imprints obtained).

To our knowledge, this is the first report showing significant differences among individuals in finger-to-finger bacterial transmission. The fact that a fraction of human individuals might maintain heavy bacterial colonization in the fingers means that they will be more efficient in cross-contamination, as inoculum size influences transmission between surfaces (Montville and Schaffner 2003), and reduces efficacy of hand disinfecting procedures (Kjoelen and Andersen 1992). We are aware of the possible consequences of our results in attributing particular risks for bacterial transmission to individual health workers or food handlers. In particular, they might have an impact on nosocomial epidemiology in the personnel involved in antinosocomial infection strategies. Because of this, our results need to be reinvestigated and confirmed with more volunteers and bacterial clones from different species, and possibly in other geographical locations. We consider that this preliminary work suggests the possibility of developing an interesting model of bacterial transmission which, if adequately standardized for intrapersonal and interpersonal transmission, could be used to measure human-to-human skin transmissibility of any bacterial species, and to adapt food processing activities as well as clinical team's activities procedures.
